# Effect of single and repeated potassium iodide applications versus glutathione on silver diamine fluoride-induced tooth discoloration in primary teeth: An in vitro comparative study

**DOI:** 10.34172/joddd.025.43876

**Published:** 2025-12-31

**Authors:** Maryam Hajiahmadi, Samira SadighBatha, Mohammad Javad Tarrahi

**Affiliations:** ^1^Department of Pediatric Dentistry, Dental Research Center, Faculty of Dentistry, Isfahan University of Medical Sciences, Isfahan, Iran; ^2^Dental Students’ Research Committee, Department of Pediatric Dentistry, School of Dentistry, Isfahan University of Medical Sciences, Isfahan, Iran; ^3^Department of Epidemiology and Biostatistics, School of Health, Isfahan University of Medical Science, Isfahan, Iran

**Keywords:** Dental caries, Glutathione, Pediatric dentistry, Potassium iodide, Tooth discoloration

## Abstract

**Background.:**

This study aimed to evaluate the effects of a single application of potassium iodide (KI), repeated daily applications of KI, and glutathione (GSH) on silver diamine fluoride (SDF)-induced tooth discoloration in primary canines.

**Methods.:**

This in vitro study was performed on 36 extracted primary canines with dentinal caries, which were randomly assigned to four treatment groups: A (38% SDF alone), B (38% SDF+immediate single application of KI), C (38% SDF+repeated daily KI applications), and D (38% SDF+20% GSH). Color changes were measured at baseline and after 1, 14, and 30 days using a spectrophotometer and the CIEL*a*b* system. Statistical analysis included one-way ANOVA with post hoc Tukey tests for inter-group comparisons, and repeated-measures ANOVA with Bonferroni’s correction for intra-group comparisons.

**Results.:**

Significant differences in tooth discoloration (ΔE) were observed between the study groups at all time intervals (*P*<0.001). Group A showed the highest discoloration, while group D exhibited the lowest. Among the KI-treated groups, group B showed progressive discoloration over time, while group C demonstrated minimal changes, with no statistically significant differences between time intervals (*P*=0.435). Although groups C and D both showed reduced discoloration compared to group B, the difference between them was not statistically significant on day 30 (*P*=0.073).

**Conclusion.:**

Repeated daily applications of KI or GSH effectively reduced SDF-induced tooth discoloration in primary canines. GSH showed the most favorable esthetic outcomes, suggesting its potential as a promising adjunct to SDF.

## Graphical Abstract

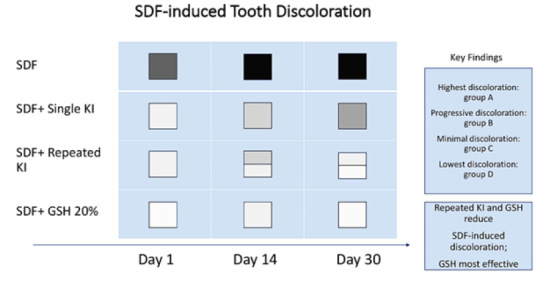

## Introduction

 Dental caries is the most prevalent chronic condition in childhood, resulting from a complex interplay of factors. It occurs due to an imbalance between demineralization and remineralization of tooth structure.^1^ Factors such as dental susceptibility, inadequate oral hygiene, and poor dietary habits increase individuals’ vulnerability to dental caries.^2^ Given the high prevalence of dental caries, preventive measures are more cost-effective than invasive restorative treatments.^3^

 Without timely intervention, demineralization progresses to involve the pulp tissue, leading to severe consequences such as pain, reduced appetite, difficulty chewing, sleep disturbances, periapical periodontitis, abscesses, and parulis in the furcation area.^4^ Consequently, untreated caries negatively affects the oral health-related quality of life of both children and their caregivers.^5^ Conventional management of dental caries involves excavation of the decayed dentin, which is followed by restorative procedures. This can be challenging due to high costs, the need for advanced dental expertise, and poor patient cooperation in young children.^6^

 In minimally invasive dentistry, preserving tooth structure is paramount.^7^ Topical fluoride agents applied directly to carious lesions can slow the progression of demineralization and convert active lesions into inactive ones.^8^

 Silver diamine fluoride (SDF) is a clear solution containing ionic silver, fluoride, and ammonia, which arrests the progression of carious lesions.^9^ Silver ions disrupt bacterial metabolism, leading to biofilm destruction, while deposition of silver salts occludes dentinal tubules. Moreover, the reaction between SDF and calcium and phosphate ions of the tooth surface forms less soluble fluorohydroxyapatite crystals. High concentrations of silver ions also inhibit matrix metalloproteinases, preventing collagen degradation and promoting remineralization.^10,11^ SDF has demonstrated superior efficacy compared to fluoride varnish in arresting active carious lesions.^12^ Additionally, SDF is used to reduce dentinal hypersensitivity, which may be important in managing teeth affected by molar-incisor hypomineralization (MIH).^13^ However, tooth staining associated with SDF limits its esthetic acceptability and clinical use.^14^

 Potassium iodide (KI) application immediately following SDF has been proposed to reduce discoloration. KI’s iodide ions react with the free silver ions responsible for black staining, forming a creamy-white precipitate that mitigates discoloration. Importantly, KI does not compromise SDF’s efficacy in enhancing enamel microhardness or antibacterial activity within dentinal tubules.^15,16^ García-Bernal et al^17^ reported greater biocompatibility of the SDF/KI combination compared to SDF alone on human exfoliated deciduous teeth stem cells (SHEDs).

 More recently, glutathione (GSH) has emerged as an alternative to KI. GSH’s thiol groups chemically reduce metal ions like silver, preserving silver ions both in SDF and on tooth surfaces.^18^ By surrounding silver particles, GSH prevents their aggregation and deposition as dark-colored compounds.^19^ Its use alongside SDF has also been shown to reduce SDF’s cytotoxicity on dental pulp cells.^20^

 Several studies have assessed the effectiveness of KI and GSH in reducing SDF-induced staining, with some comparing the two agents directly. However, most employed a single application protocol. To investigate whether application frequency influences KI’s efficacy, the present study included a group receiving daily repeated KI applications following SDF treatment. This study compared the effectiveness of single-application KI, repeated daily KI applications, and GSH in reducing tooth discoloration caused by 38% SDF in primary canines.

## Methods

###  Study Design and Sample Collection

 This in vitro study was conducted on extracted primary canines with dentinal carious lesions and no previous restorations, collected from the Pediatric Dentistry Department of Isfahan University of Medical Sciences. All the teeth had been extracted within the past 6 months. The study protocol was approved by the Ethics Committee of Isfahan University of Medical Sciences (Approval Code: IR.MUI.DHMT.REC.1403.186)

 The sample size was calculated using G*Power software (version 3.1.9.7) based on a previous study, with a significance level of 5% (α = 0.05) and a study power of 80% (1-β = 0.8).^21^ According to this calculation, 8 specimens per group were sufficient to achieve adequate statistical power; we included 9 specimens per group, slightly exceeding this requirement. A total of 36 primary canines were included, meeting the following inclusion criteria:

###  Inclusion Criteria

- Extracted primary canines with carious lesions involving both enamel and dentin
- Teeth free from developmental anomalies

###  Exclusion Criteria

- Cracked or fractured teeth
- Previously restored teeth
- Teeth with arrested caries or dark-colored carious lesions
- Endodontically treated teeth.

###  Specimen Preparation

 All 36 teeth were stored in distilled water to prevent dehydration and later immersed in 0.5% sodium hypochlorite for 7 days for disinfection. The teeth were then mounted in acrylic resin up to 1 mm below the cementoenamel junction (CEJ) and aligned perpendicular to their longitudinal axis. Partial removal of carious tissue was performed manually using a hand instrument. The specimens were then randomly assigned to four groups (n = 9) using a random numbers table:

- Group A: 38% SDF
- Group B: 38% SDF + single-application of KI
- Group C: 38% SDF + repeated daily application of KI
- Group D: 38% SDF + GSH.

###  Intervention Protocols

 All the materials were applied according to the manufacturers’ instructions.

- Group A: 38% SDF (Fagamin, Tedequim, Argentina) was applied to the tooth surface using a microbrush, agitated for 1 minute, left undisturbed for 2 minutes, and then rinsed with water for 30 seconds.
- Group B: 38% SDF was followed by an immediate application of a saturated KI solution (Rivastar, SDI, Australia) until the creamy-white precipitate cleared, after which the surface was rinsed with water for 30 seconds.
- Group C: Similar to group B, followed by daily application of saturated KI solution for 10 consecutive days.
- Group D: 38% SDF (Fagamin, Tedequim, Argentina) was mixed with 20 wt% of GSH (Merck, Germany) under vigorous stirring until a transparent, precipitate-free solution was obtained, which was applied in the same manner as group A.

All the specimens were restored individually in artificial saliva in sealed glass containers and incubated at 37 °C.

###  Color Assessment

 Color measurements were taken at baseline (before treatment), and on days 1, 14, and 30 after treatment. A spectrophotometer (Shadepilot, Degudent; Hanau-Wolfgang, Germany) was used, operating within a 400–700 nm wavelength range. The device was calibrated before each use according to the manufacturer’s instructions. Color was recorded using the CIEL*a*b* color system, where L* represents brightness (0 = dark, 100 = bright), a* indicates ranges from green (-a) to red ( + a), and b* describes the blue (-b) to yellow ( + b) spectrum. Each measurement was repeated three times per specimen, and the mean value was used. The overall color change (ΔE) was calculated using the following formula:

 ΔE = [(ΔL)^2^ + (Δa)^2^ + (Δb)^2^]^1/2^

###  Statistical Analysis

 Data normality was assessed using the Shapiro–Wilk test. Inter-group comparisons at each time interval were performed using one-way ANOVA with post hoc Tukey tests. Intra-group comparisons over time were assessed using repeated-measures ANOVA with Bonferroni’s post hoc correction. To evaluate the main effects of time, treatment, and their interaction, a two-way repeated-measures ANOVA was conducted. A *P* < 0.05 was considered statistically significant.All the analyses were performed using SPSS 26 (IBM Corp., Armonk, NY, USA).

## Results

###  Inter-Group Comparisons

**Figure 1:**
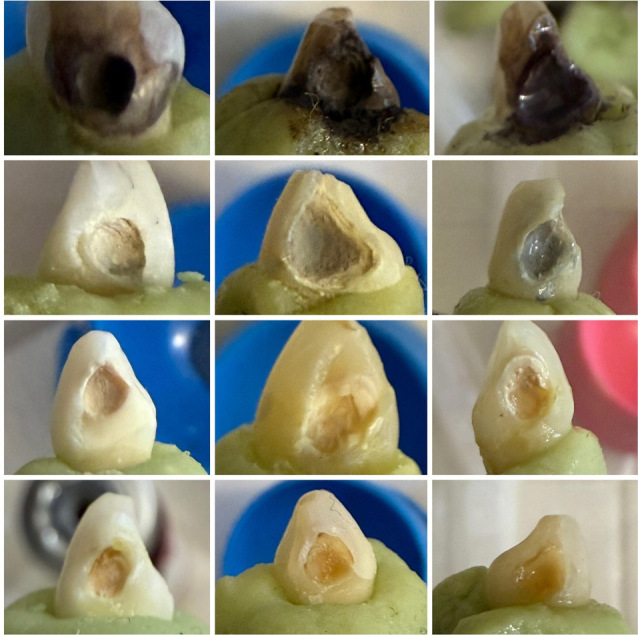
Representative samples from each experimental group on days 1, 14, and 30 after intervention. From top to bottom: group A (SDF alone), group B (SDF + immediate single KI application), group C (SDF + repeated daily KI applications), and group D (SDF + GSH). From left to right on days 1, 14, and 30.

Table 1 presents the mean values ± standard deviations for color change (∆E) values for each group at different time intervals. Group A (SDF alone) exhibited the highest ∆E values at all time intervals, while group D (SDF + GSH) showed the lowest values. It should be noted that higher ∆E values indicate greater discoloration and darker tooth colors. Figure 1 presents representative images of treated teeth from each group on days 1, 14, and 30.

**Table 1:** Mean ± SD of color change (∆E) values on days 1, 14, and 30 for each group, and the results of one-way ANOVA for intervention, repeated-measures ANOVA for time, and two-way repeated-measures ANOVA for interactive effects

**Group**	**N**	**Day 1** **Mean±SD**	**Day 14** **Mean±SD**	**Day 30** **Mean±SD**	***P*** ** value intervention**	***P*** ** value interaction**	***P*** ** value time**
SDF	9	32.4411 ± 3.52076	40.0156 ± 5.10752	40.3767 ± 4.92264	0.000	0.000	0.002
SDF + single KI	9	11.0833 ± 1.83989	14.7700 ± 2.23628	26.0922 ± 4.16239			0.000
SDF + repeated daily KI	9	10.8322 ± 1.38356	11.3233 ± 3.76859	12.1678 ± 4.09288			0.435
SDF + GSH	9	6.4278 ± 2.91991	10.0200 ± 2.63514	7.2278 ± 3.13619			0.002
*P* value		0.000	0.000	0.000			

 Post hoc pairwise comparisons revealed the following:

- On day 1, all the groups differed statistically from each other (*P* < 0.05), except for group B (SDF + single KI) and group C (SDF + repeated daily KI) (*P* = 0.997).
- On day 14, all pairwise comparisons were significantly different, except between groups B and C (*P* = 0.201), and groups C and D (SDF + GSH) (*P* = 0.869).
- On day 30, significant differences were observed between all the groups, except between groups C and D (*P* = 0.073).

Table 2 summarizes the detailed results of intergroup comparisons.

**Table 2:** Post hoc pairwise comparisons of color changes (∆E) between treatment groups on days 1, 14, and 30

**Comparison**	**Day 1** ***P*** ** value**	**Day 14** ***P*** ** value**	**Day 30** ***P*** ** value**
SDF vs. (SDF + single KI)	< 0.001	< 0.001	< 0.001
SDF vs. (SDF + repeated daily KI)	< 0.001	< 0.001	< 0.001
SDF vs. (SDF + GSH)	< 0.001	< 0.001	< 0.001
(SDF + single KI) vs. (SDF + repeated daily KI)	0.997	0.201	< 0.001
(SDF + single KI) vs. (SDF + GSH)	0.003	0.042	< 0.001
(SDF + repeated daily KI) vs. (SDF + GSH)	0.005	0.869	0.073

###  Intra-Group Comparisons

 As shown in Table 1, repeated-measures ANOVA revealed statistically significant differences in ΔE values over time in all the groups (*P* < 0.05), except for group C (SDF + repeated daily KI) (*P* = 0.435).

 Pairwise comparisons across the three time intervals in each group revealed the following:

- In group A (SDF alone), tooth discoloration significantly increased from day 1 to days 14 and 30 (*P* < 0.05), with no significant difference between days 14 and 30.
- In group B (SDF + single KI), ΔE values progressively increased, with significant differences between all three time intervals (*P* < 0.001).
- In group C (SDF + repeated daily KI), no significant differences in color change were observed between any of the time intervals (*P* > 0.05).
- In group D (SDF + GSH), ΔE values on days 1 and 30 were significantly lower than on day 14, while no significant difference was found between days 1 and 30 (*P* = 1).

Table 3 provides detailed intra-group post hoc comparisons.

**Table 3:** Pairwise comparisons of color changes (∆E) across time intervals in each group

**Group**	**Time comparison**	***P*** ** value**
SDF	Day 1 vs. Day 14	0.007
SDF	Day 1 vs. Day 30	0.005
SDF	Day 14 vs. Day 30	0.636
SDF + single KI	Day 1 vs. Day 14	< 0.001
SDF + single KI	Day 1 vs. Day 30	< 0.001
SDF + single KI	Day 14 vs. Day 30	< 0.001
SDF + repeated daily KI	Day 1 vs. Day 14	1.000
SDF + repeated daily KI	Day 1 vs. Day 30	0.766
SDF + repeated daily KI	Day 14 vs. Day 30	1.000
SDF + GSH	Day 1 vs. Day 14	0.011
SDF + GSH	Day 1 vs. Day 30	1.000
SDF + GSH	Day 14 vs. Day 30	0.020

###  Interactive Effect of Time and Treatment 

 A statistically significant interaction between time and intervention type (*P* = 0.002) was found, indicating that the pattern of color change over time varied across treatment groups. In other words, the rate and direction of discoloration progression differed between the groups across the three time intervals.

## Discussion

 SDF is widely recognized as a practical, affordable, and noninvasive cariostatic agent, particularly for young children.^22,23^ Two randomized clinical trials have demonstrated that 38% SDF is more effective than 12% for caries control,^24,25^ supporting the use of the higher concentration in this study. Its high levels of fluoride (6%; 44,880 ppm) and silver (25.5%) ions react with hydroxyapatite, forming calcium fluoride and silver phosphate precipitates. These compounds not only possess antimicrobial properties but also physically occlude dentinal tubules, contributing to SDF’s desensitizing and protective effects. CaF_2_ acts as a fluoride reservoir to promote fluorohydroxyapatite formation, while Ag_3_PO_4_ crystals—initially yellow—darken upon exposure to light or oxidizing agents. Additionally, free silver ions (Ag^+^) are reduced to black precipitates, leading to the characteristic discoloration associated with SDF application.^18,26-28^ To address this esthetic drawback, adjunctive agents such as KI and GSH have attracted interest. This in vitro study evaluated the efficacy of KI, including both single and repeated applications, and GSH in minimizing SDF-induced tooth discoloration in primary canines.

 KI is believed to react with free silver ions to form a white silver iodide precipitate (AgI), thereby preventing the formation of darker silver compounds.^18,29^ While prior studies generally agree on the short-term benefits of KI in reducing SDF-related staining, most have used single application protocols, and their long-term results remain inconsistent.^21,30,31^

 In the present study, single-application KI significantly reduced discoloration on days 1 and 14, but its effect diminished by day 30, suggesting a limited long-term benefit. In contrast, repeated KI applications maintained significantly lower discoloration levels across all time intervals. Notably, only the group receiving repeated KI for 10 days (group C) showed no significant intra-group color change over time, likely due to the sustained availability of iodide ions, which neutralize free silver. Conversely, the single-application KI group (group B) showed progressive darkening, presumably because declining iodide levels allowed residual free silver ions to precipitate over time.

 These findings are supported by Hamdy et al,^32^ who showed that a single application of KI effectively masked initial SDF discoloration, though its long-term durability remained uncertain. Similarly, Detsomboonrat et al^30^ reported that KI had a dose-dependent masking effect immediately after application, but this benefit waned after 14 days, with no significant differences between different concentrations. These findings suggest that even high KI concentrations may not maintain their esthetic effect over time unless applied repeatedly.

 Despite promising outcomes with repeated KI use, a limitation of the present study is the daily application protocol over 10 consecutive days, which may be impractical in clinical settings. Future studies should investigate optimized regimens, such as reducing the number of application days or using alternate-day applications to balance feasibility and effectiveness.

 GSH, a thiol-containing antioxidant, chelates and stabilizes silver ions, reducing their aggregation and enhancing solubility in aqueous environments.^19^ This mechanism may contribute to more stable esthetic results.

 In the present study, 20% GSH provided the most favorable esthetic outcome across all time intervals, showing a statistically significant superiority over both groups A (SDF alone) and B (SDF + single application KI), and comparable efficacy to repeated KI application (group C).

 Based on the available literature, six studies have investigated GSH as a stain-mitigating agent in comparison with single-application KI.^18,21,31,33-35^ Our findings partially align with Karuna et al,^31^ who in a clinical split-mouth trial reported better immediate results with single-application KI but superior long-term esthetics with 20% GSH at the 6-month follow-up. Samani et al,^33^ assessed esthetic changes only on day 1 and found no significant differences between single-application KI and GSH, though GSH performed slightly better.

 In contrast, Cömert et al^34^ found that 5% GSH showed no benefit compared with SDF alone, whereas single-application KI improved immediate esthetics. The inconsistency with our findings may be attributed to the lower GSH concentration and shorter observation period used in their study.

 Kamble et al^21^ reported favorable esthetic outcomes for both 20% GSH and single-application KI over 30 days in vitro, with slightly better results at later stages for single-application KI. Unlike our study, they did not observe a decline in single-application KI effectiveness over time. Their use of glass-ionomer restorations over the treated lesions may contribute to this discrepancy. This may also explain the lower ΔE values compared to our study.

 Sayed et al,^18^ in an in vitro study on bovine teeth, observed comparable early results for single-application KI and GSH, with single KI outperforming GSH. They also noted greater efficacy of both agents in enamel lesions than in dentin lesions. Similarly, Gupte et al,^35^ who evaluated artificial enamel caries, observed that while the esthetic effects of GSH and single-application KI were comparable up to 24 hours, KI demonstrated superior performance over a one-month follow-up.

 Taken together, while single-application KI provides initial esthetic improvements by binding free silver ions, the limited iodide availability may be insufficient to neutralize residual ions within dentinal tubules in the long term, leading to progressive discoloration. In contrast, GSH appears to exert a more stable effect through silver ion chelation. Repeated KI application helps maintain iodide availability and enhance long-term esthetic outcomes. Notably, this study used naturally carious primary canines with dentin involvement, a more clinically relevant model than artificially induced caries models.^36^ This aligns with real-world application, as SDF is more commonly used for advanced cavitated lesions rather than early non-cavitated ones.^37^

 This study contributes to the literature by directly comparing repeated KI applications and 20% GSH, addressing the current gap in evidence regarding outcomes of repeated KI protocols.

 Finally, multiple factors such as KI application frequency, GSH concentration, lesion depth, light exposure, and potentially even patient-related variables (e.g., oral hygiene or dietary habits) may influence esthetic outcomes. Further long-term clinical trials evaluating various frequencies and concentrations under real-world conditions are warranted to refine application protocols for these adjunctive agents and achieve therapeutic efficacy with acceptable esthetics.

 This study has some limitations that should be acknowledged. First, the follow-up period was limited to 30 days, which was chosen based on previous in vitro studies investigating SDF-induced discoloration to maintain consistency with the literature. Extending the observation interval was not feasible due to laboratory scheduling, costs, and potential environmental changes that could affect the specimens. Second, restorations were not placed after treatment in order to accurately evaluate intrinsic tooth discoloration; while this ensured precise colorimetric assessment, it does not fully replicate the clinical scenario and should be addressed in future in vivo studies. Finally, the sample size was relatively limited; however, G*Power calculations indicated that 8 specimens per group would be sufficient to achieve adequate statistical power. We included 9 specimens per group, slightly exceeding the required number. Although the inclusion criteria required teeth with dentin caries, a sufficient number of specimens were available to detect statistically significant differences between groups. Future studies with longer follow-up periods and larger sample sizes in clinical settings are recommended to further validate these findings.

## Conclusion

 Within the limitations of this in vitro study, it can be concluded that while SDF causes significant tooth discoloration, the adjunctive use of KI and GSH can mitigate this undesirable effect. A single application of KI resulted in a moderate reduction in staining, whereas repeated daily KI applications provided greater color stability over time. GSH showed the most favorable esthetic outcomes throughout the study period. Therefore, either repeated KI application or GSH can be considered promising adjuncts to SDF where esthetics is a concern.

## Competing Interests

 The authors declare no conflicts of interest.

## Ethical Approval

 The study was approved by the Research Ethics Committee of Isfahan University of Medical Sciences under the code IR.MUI.RESEARCH.REC.1402.192.
